# Mechanical Performance of 3D-Printed Resins versus CAD/CAM PMMA for Provisional Crowns: New Evidence under Simulated Clinical Conditions

**DOI:** 10.4317/jced.63128

**Published:** 2025-10-01

**Authors:** Aracy Diana Zarate-Maquera, Marco Sánchez-Tito, José Giancarlo Tozo-Burgos

**Affiliations:** 1 Research Group on Dental Biomaterials and Natural Products, Faculty of Health Sciences, Universidad Privada de Tacna, 23000 Tacna, Peru

## Abstract

**Background:**

Provisional restorations play a fundamental role in fixed prosthodontic rehabilitation. While current evidence has identified CAD/CAM-fabricated materials as the preferred option due to their excellent mechanical properties, 3D-printed resins have shown significant improvements in their performance in recent years. Therefore, the aim of the present study was to re-evaluate the fracture resistance of provisional crowns fabricated using 3D-printed resin and CAD/CAM-milled PMMA, considering the influence of artificial aging.

**Material and Methods:**

An *in vitro* study was conducted on 60 provisional crowns divided into four groups according to material type (3D-printed resin or CAD/CAM PMMA) and aging condition; thermocycling and simulated brushing were applied, fracture resistance was tested using a universal testing machine, and data were analyzed using the Student’s t-test at a 5% significance level.

**Results:**

After artificial aging, 3D-printed restorations showed significantly higher fracture resistance than CAD-CAM milled crowns (*p* = 0.0064). However, no statistically significant differences were observed between the two fabrication methods under non-aged conditions (*p* > 0.05). All groups exceeded the minimum values considered clinically accepTable.

**Conclusions:**

3D printing demonstrated superior mechanical stability after artificial aging, supporting its clinical viability as an efficient, predicTable, and favorable option for provisional restorations in oral rehabilitation.

** Key words:**3D printing, CAD/CAM, Digital dentistry, Provisional restoration, fracture resistance.

## Introduction

Provisional restorations are essential components in fixed prosthodontic rehabilitation, as they preserve function, aesthetics, and the integrity of oral tissues during the transitional phase preceding the placement of the definitive prosthesis [1]. Their omission or inadequate fabrication can lead to clinical complications such as postoperative hypersensitivity, pulpitis, or periodontal damage [2].

In complex clinical situations, such as full-mouth rehabilitations, occlusal stabilization treatments, or cases involving severely worn dentition, provisional restorations may remain intraorally for extended periods [1,3,4]. In these scenarios, materials with appropriate physical and mechanical properties are required to ensure optimal clinical performance. Polymethyl methacrylate (PMMA) has traditionally been widely used due to its biocompatibility, ease of manipulation, and low cost; however, its mechanical behavior may be compromised when fabricated using conventional polymerization techniques [5].

The integration of digital technologies has led to significant improvements in the fabrication of provisional restorations. CAD/CAM milling, 3D printing, and both direct and indirect digital impression techniques have enhanced marginal adaptation, reduced chairside time, and enabled the use of materials with superior mechanical properties [6-8]. CAD/CAM PMMA blocks possess a highly cross-linked polymer structure that improves fracture resistance and dimensional stability while reducing porosity [9]. In contrast, 3D-printed resins, typically fabricated using stereolithography (SLA) or fused deposition modeling (FDM), exhibit a layered microstructure that may compromise mechanical integrity. However, recent advances in their formulation, particularly the incorporation of inorganic fillers such as silica or zirconia, have resulted in substantial improvements in mechanical performance [8,10,11].

Several studies have demonstrated that provisional crowns fabricated from CAD/CAM-milled PMMA exhibit higher fracture resistance compared to their 3D-printed counterparts, although the latter remain within clinically accepTable limits [12]. In the case of three-unit fixed dental prostheses, milled restorations also show superior mechanical performance, but 3D-printed alternatives may be considered a viable option in areas of the arch with lower occlusal demands [13].

Fracture resistance testing is one of the most commonly used methods to evaluate the mechanical behavior of dental materials. It typically involves the application of compressive load until structural failure [14-16]. However, to obtain clinically relevant results, artificial aging protocols such as thermocycling, cyclic loading, and simulated toothbrushing must be applied, as these simulate the degradative effects of the oral environment [17,18].

Despite technological advances, the literature on the mechanical performance of materials specifically designed for provisional restorations remains limited. In this context, the present study aimed to compare the fracture resistance of provisional crowns fabricated from 3D-printed resin and CAD/CAM-milled PMMA using artificial aging protocols that simulate intraoral conditions.

## Material and Methods

-Study design and sample size calculation.

This *in vitro* study was approved by the Ethics Committee of Universidad Privada de Tacna (FACSA-CEI/070-04-2024). The sample size was determined using G*Power 3.1.3 software, applying an ANOVA test with a significance level of α = 0.05, statistical power of 80%, and an effect size of 0.40, resulting in a minimum of 51 specimens. To ensure proper distribution and account for potential losses, the final sample size was increased to 60 specimens, distributed into four groups according to material type and aging condition. The groups labeled CAD-CAM AA and 3D AA were subjected to artificial aging, while the CAD-CAM and 3D groups did not undergo this process.

The eligibility criteria for the specimens were based on proper fabrication, ensuring that they were intact and exhibited uniform dimensions. As exclusion criteria, specimens were discarded if they exhibited fractures, cracks, delamination, printing or milling defects, visible contamination, dimensional deviations from the established parameters, or if they had undergone incorrect curing or aging procedures.

-Manufacturing and Preparation of Specimens

For the design of the provisional crowns, a dental preparation performed on a conventional typodont was used as a reference. This typodont was scanned using the S600 ARTI scanner (Zirkonzahn, South Tyrol, Italy), and the resulting digital file was processed with the Zirkonzahn.Modellier design software. Based on this design, a master mold was milled in Titanit (Zirkonzahn, South Tyrol, Italy) using the M5 Heavy Metal milling unit (Zirkonzahn, South Tyrol, Italy).

**Figure 1:**
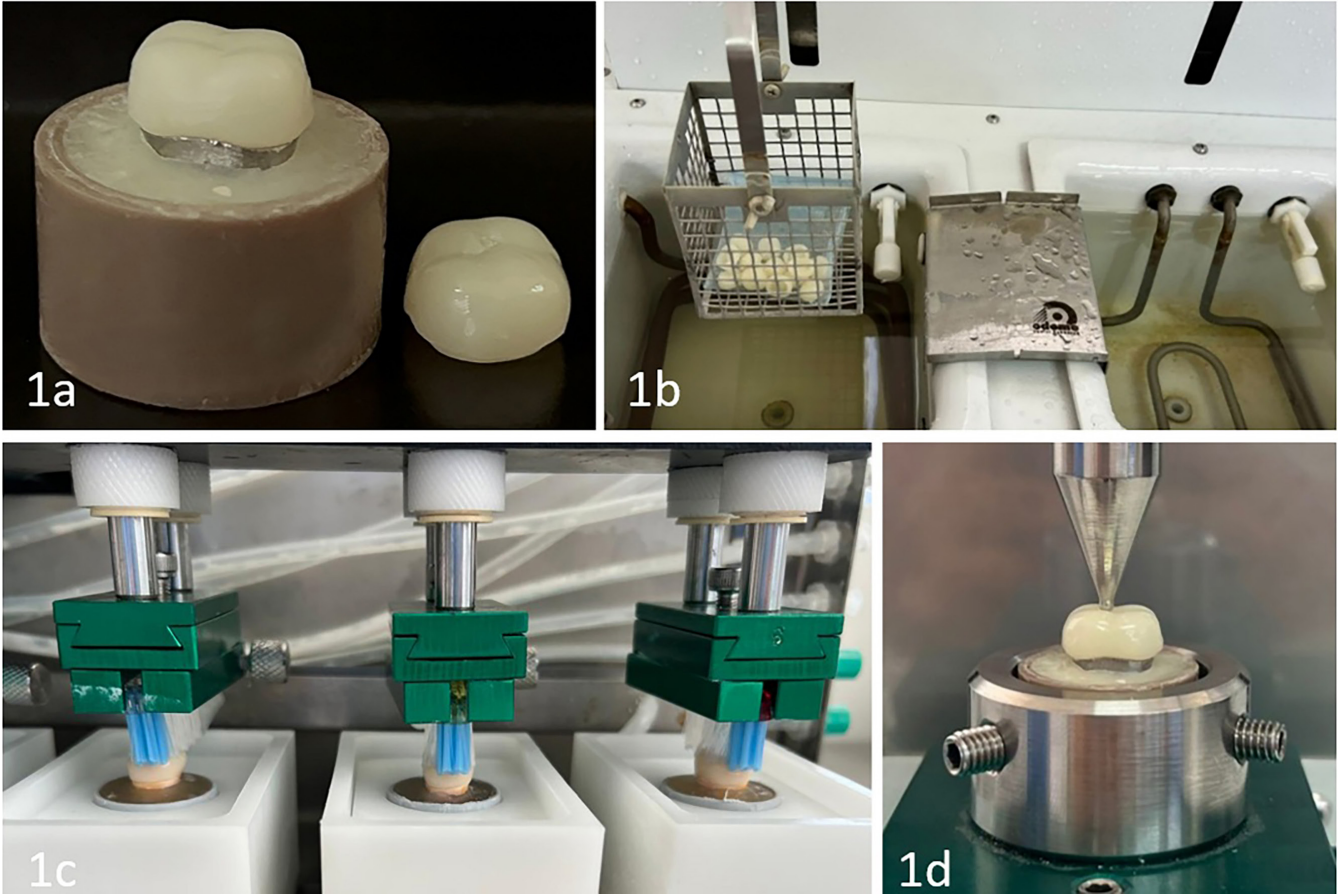
Experimental procedure, (1a) provisional crown specimen, (1b) thermocycling protocol, (1c) simulated toothbrushing, (1d) fracture resistance testing procedure.

Using the master typodont, the provisional crowns were designed with the same software, defining a uniform thickness of 2 mm. One group was fabricated in PMMA Provisional 95 by milling with the Milling Unit M1 (Zirkonzahn, South Tyrol, Italy). The corresponding STL file was also imported into an AccuFab-D1s 3D printer (Shining 3D, Hangzhou, China) to fabricate another group of crowns using PriZma 3D Bio Prov resin (Makertech Labs, Maringá, Brazil), in accordance with the manufacturer’s instructions (Fig. 1a).

-Simulation of Clinical Aging

One group of specimens was subjected to a thermocycling protocol using the OMC350 TS unit (Odeme Dental Research, Joaçaba, Brazil), programmed to perform 5000 cycles between extreme temperatures of 5 °C and 55 °C. Each cycle was conducted in distilled water, with an immersion time of 25 seconds in each thermal bath. (Fig. 1b).

Subsequently, the specimens were exposed to a toothbrushing simulator (Odeme Dental Research, Joaçaba, Brazil), equipped with eight toothbrush heads arranged in the device’s chambers. A mixture of toothpaste (Colgate®) and distilled water in a 1:2 ratio was used. The protocol consisted of 5000 brushing cycles, at a speed of 30 cycles per minute, with a vertical load of 200 grams applied (Fig. 1c).

-Fracture Resistance Test

Fracture resistance was evaluated using a universal testing machine (OM 150, Odeme Dental Research, Joaçaba, SC, Brazil). The specimens were centered on the testing platform and subjected to an axial compressive load applied by a 5 mm diameter steel spherical piston. The load was continuously increased at a crosshead speed of 1 mm/min, applied directly to the central fossa of each restoration until fracture occurred. The load required to induce fracture was recorded in Newtons (N) [12] (Fig. 1d).

-Statistical Analysis

Data were analyzed using Stata® software version 17 (StataCorp LP, College Station, TX, USA); error bar graphs were constructed using GraphPad Prism version 10.0.0 (GraphPad Software, Boston, Massachusetts, USA). Normality and homoscedasticity were assessed using the Shapiro–Wilk test and the Variance ratio test, respectively. Student’s t test was used to compare fracture toughness between groups. A significance level of 5% was adopted for all tests.

## Results

 Table 1 presents the descriptive statistics of fracture resistance (mean ± standard deviation) for CAD-CAM and 3D-printed specimens, evaluated with and without artificial aging. The 3D-printed groups showed higher mean fracture resistance values than their CAD-CAM counterparts across both conditions. The highest mean was observed in the non-aged 3D group (557.89 ± 55.74 N), followed closely by the aged 3D group (555.64 ± 43.47 N), suggesting a limited effect of aging on 3D-printed materials. Conversely, the CAD-CAM groups demonstrated lower values overall, with a more noticeable decrease in the aged subgroup (503.26 ± 53.37 N) compared to the non-aged group (519.53 ± 75.81 N).

**Figure 2:**
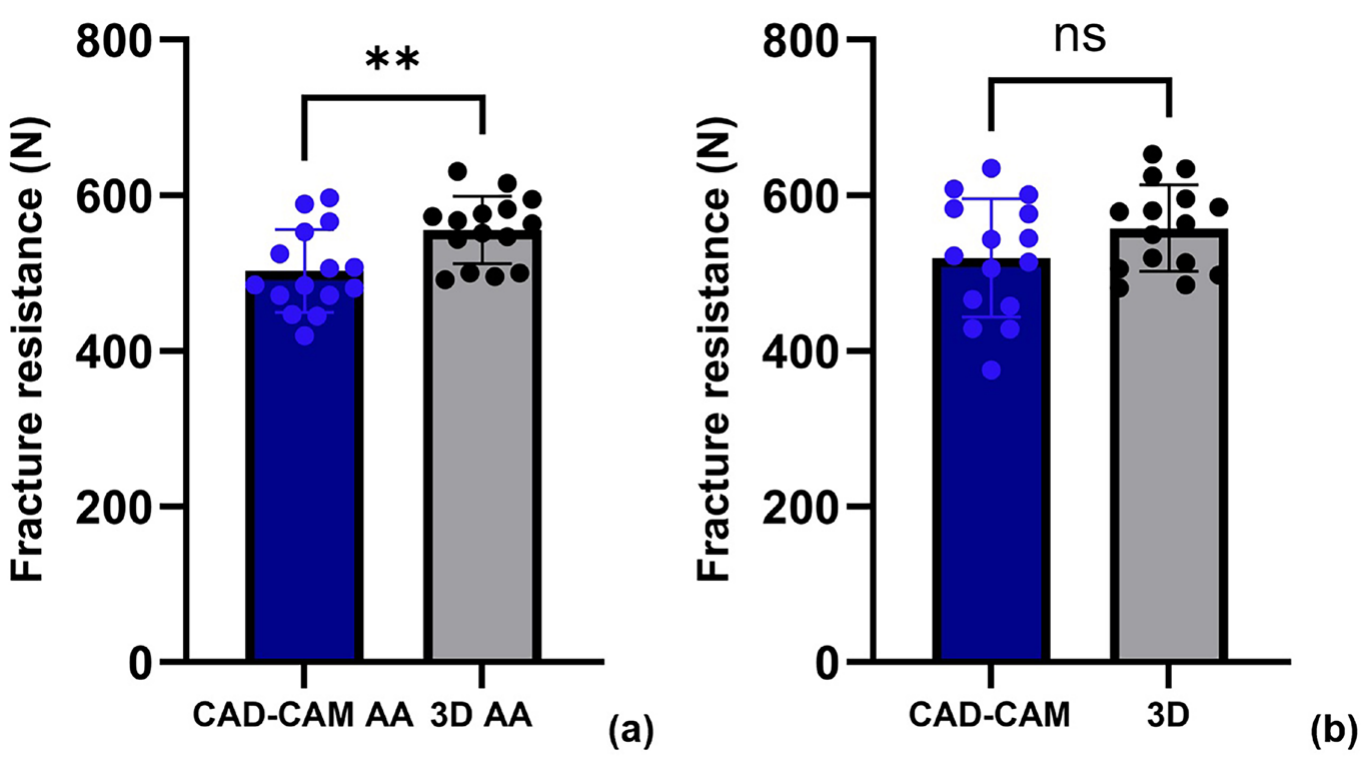
Fracture resistance of CAD-CAM and 3D-printed specimens with and without artificial aging, (a) Comparison of fracture resistance between CAD-CAM and 3D printed specimens after artificial aging, (b) Comparison without artificial aging. **statistically significant difference (*p* = 0.0064), ns: *p* > 0.05.

As shown in Fig. 2, specimens fabricated using the 3D printing technique exhibited significantly higher fracture resistance after artificial aging compared to those produced by the CAD-CAM method (*p* = 0.0064). In contrast, no statistically significant difference was observed between the two fabrication methods in the absence of artificial aging (*p* > 0.05).

## Discussion

The present study evaluated the fracture resistance of two materials commonly used during the provisionalization phase of oral rehabilitation. The comparison focused on their mechanical behavior, specifically their fracture resistance. Until recently, scientific evidence has favored CAD/CAM-milled materials over those fabricated through 3D printing. This presumed superiority is attributed to the PMMA blocks used in CAD/CAM systems, which are industrially pre-polymerized. This process results in high monomer conversion, low porosity, and a homogeneous microstructure. In contrast, 3D-printed materials are created layer by layer, a characteristic that has historically been considered a potential risk factor for mechanical performance [19].

A recent study conducted by Abad-Coronel *et al*. [12] utilized a methodology similar to the one used in this research. The study involved a metallic master typodont and applied the same number of thermocycling cycles while designing crowns with comparable characteristics. It evaluated the fracture resistance of single-unit crowns fabricated using both digital techniques, finding that milled restorations exhibited higher resistance. Similarly, another study that investigated the fracture resistance of three-unit provisional fixed prostheses also reported better mechanical performance in milled restorations [13], which aligns with the conclusions of a previously mentioned systematic review [19].

Despite following a similar methodological approach, the results of this study differed significantly from previous findings. The 3D-printed provisional crowns demonstrated notably higher fracture resistance and better preservation of structural integrity when subjected to simulated clinical conditions, including an artificial aging protocol with thermocycling and brushing cycles. Additionally, these procedures had a more pronounced impact on CAD/CAM-milled restorations. This new evidence may be attributed to recent advancements in 3D printing resin formulations, which now include inorganic fillers like silica, zirconia, or glass to enhance mechanical strength, translucency, and thermal dimensional stability [20].

The resin used in this study was PriZma 3D Bio Prov, for which no detailed information about its internal composition has been made publicly available by the manufacturer. However, a previous study that analyzed various 3D-printed resins for provisional restorations included this material. Although the primary objective of that study was to evaluate the effect of build orientation, a complementary microscopic analysis was performed using scanning electron microscopy with energy-dispersive X-ray spectroscopy (SEM-EDS). This analysis revealed that PriZma Bio Prov contained very few spherical nanometric filler particles dispersed throughout its organic matrix.

This characteristic suggests that the material has a more fluid and homogeneous formulation, which enhances dimensional accuracy, surface finish, and print quality—features that are particularly relevant for 3D-printed provisional restorations. However, the low concentration of reinforcing fillers may limit its absolute mechanical strength when compared to other resins with a higher inorganic filler load [21].

Despite this, the results obtained in the present study demonstrated a mechanical performance superior to that reported in previous investigations, which could be attributed to improvements in the resin’s formulation or the use of more controlled printing and post-curing parameters. This improvement aligns with the current trend in the development of next-generation 3D printing resins, which are being reformulated with cross-linked multifunctional monomers and inorganic fillers, enabling a high degree of polymer conversion and, consequently, enhanced mechanical properties [22]. Although specific compositional details of PriZma Bio Prov have not been disclosed, its observed performance is consistent with this ongoing evolution in 3D printing materials.

It is important to highlight the clinical significance of the artificial aging protocol employed, which included thermocycling and simulated tooth brushing, simulating approximately two years of oral function. This method is relevant clinically because the lifespan of a provisional restoration rarely exceeds this timeframe. During this period, material degradation and functional stress can weaken the organic matrix, leading to the development and spread of microcracks in porous areas. These structural changes may result in fractures or adhesive failures, particularly after the first year of clinical use [23].

he use of simulated clinical conditions allowed for a more realistic evaluation of the effects of aging on provisional crowns. In this context, 3D-printed restorations demonstrated higher fracture resistance compared to milled ones, both before and after artificial aging, with mean values of 557.89 N for 3D-printed crowns and 555.64 N for milled crowns, indicating minimal degradation. In contrast, the CAD/CAM group exhibited a more significant reduction in resistance, decreasing from 519.53 N to 503.26 N, highlighting a greater susceptibility to the effects of aging. This difference may be attributed to the fact that milling can generate structural micro-defects, which can induce internal stress or microcracks, particularly in thin or geometrically complex areas [24].

To date, only one study has shown that 3D printing systems are superior to other techniques for fabricating provisional restorations. In this study, Alageel *et al*. [17] evaluated the physical and mechanical properties of provisional resins produced using three different methods: a conventional technique, CAD/CAM milling, and 3D printing. They applied an artificial aging protocol similar to the one used in the present study. The results indicated a significant decrease in microhardness across all groups; however, the 3D-printed group maintained the highest microhardness values. Additionally, the flexural strength of the 3D-printed resins was consistently higher than that of the other methods, both before and after aging. These findings are consistent with the results of the present study and reinforce the idea that 3D-printed resins can maintain their mechanical performance over time, establishing them as a reliable clinical alternative.

The fracture resistance values obtained for both materials are within the clinically acceptable range for temporary restorations. In healthy adults, posterior masticatory forces typically range from 424 N to 630 N, with higher values usually found in males [25]. However, in patients with parafunctional habits like bruxism, these forces can significantly exceed this range [26], which may limit the applicability of these materials due to an increased risk of fracture.

Therefore, the findings of this study suggest that 3D-printed resin is a viable and competitive clinical alternative to CAD/CAM-milled materials for provisional restorations. This provides new evidence supporting the optimization of its mechanical properties.

One main limitation of this study is the limited information available on the evaluated 3D-printed resin, which required reliance on previous studies that describe its performance and structural improvements over time. Additionally, the *in vitro* design poses a limitation as it does not fully replicate conditions in the mouth. However, all necessary precautions were taken to accurately simulate the oral environment, allowing for a realistic assessment of the materials’ behavior under clinically relevant conditions.

## Conclusions

3D-printed restorations showed significantly higher fracture resistance compared to those made through milling, even after undergoing artificial aging. These results highlight notable improvements in the structural integrity and functional performance of printed materials. However, both fabrication methods—CAD/CAM milling and 3D printing—demonstrated clinically accepTable mechanical behavior for use in temporary restorations. Overall, these findings support the clinical viability of 3D printing as an efficient, reliable, and advantageous option for provisionalization in oral rehabilitation.

## Figures and Tables

**Table 1:** Fracture resistance (N) for CAD-CAM and 3D-Printed Specimens with and without artificial aging.

Group	Media ± SD	Min	Max
CAD-CAM AA	503.26 ± 53.37	419.44	596.82
CAD-CAM	519.53 ± 75.81	375.34	635.04
3D AA	555.64 ± 43.47	491.96	631.12
3D	557.89 ± 55.74	481.18	652.68

## Data Availability

The datasets used and/or analyzed during the current study are available from the corresponding author.
